# Study on the algorithm of fault recording analysis combining its time-domain waveforms with phase-domain trajectories

**DOI:** 10.1038/s41598-024-54739-w

**Published:** 2024-02-23

**Authors:** Qun Ge, Lu Ren, Jia Li

**Affiliations:** School of Electrical and Control Engineering, Liaoning Technical University, Huludao Liaoning, 125105 China

**Keywords:** Engineering, Electrical and electronic engineering

## Abstract

The untimely handling of faults in a power system has a negative impact on its operation and even the national economy, and this requires coordination in the functions of protective relaying as well as supervisory & control devices, where digital fault recorders are used to record fault waveforms of electrical physical quantities. The fault recording of a simulated current is taken as the research object in this article, and it is transformed from the time-domain waveform into a phase-domain trajectory, which is used to analyze fault feature parameters and then reformulate the waveform. The original waveform of the current will be substituted by the reformulated one with fault features to realize functions in the power system. The algorithm of reformulating fault recording, the correlativity of the reformulated waveform and its original one, and errors produced in the research process are researched. The high correlation coefficient between the reformulated waveform and its original one shows that the algorithm studied in the article offers a simple and convenient option for fault recording analysis.

## Introduction

The development of the national economy is inseparable from sustainability of electrical energy. In a regional interconnected power system, the untimely handling of faults runs a high risk of a large-scale blackout, and it is significant to coordinate the functions of protective relaying and supervisory & control to secure the safe and reliable operation of the system^1,2^. In fault recording from the digital devices such as fault recorders or microprocessor-based protection there contained electrical physical quantities reflecting the abnormal or fault states of a power system^3–5^, which are applied to performance analysis of devices, fault detection, and protective relay setting^6^ etc. In fault waveforms of current and voltage there are fundamental frequency components, dc components, harmonics, and inter-harmonics^7^. The fault feature parameters, containing the angular frequency, amplitude, and initial phase angle in a fundamental frequency component, the initial value and time constant in a decaying dc component, the angular frequencies, amplitudes, and initial phase angles in harmonics and inter-harmonics, are accessible only after they are extracted from the waveforms.

Fault recording analysis is hitherto conducted from three aspects: time-domain analysis, analysis combining time with frequency domain, and analysis using space vectors.

In the time-domain analysis there is a solution where the eigenvalues of time-sequence features are calculated to identify the sections of single-phase-to-ground faults, and the distribution characteristics of the eigenvalues are applied to fault identification and classification by an improved K-means clustering algorithm^8^. There is also a way where fault detection and classification are completed by the voltage signals at the ends of transmission lines based on a Euclidean distance measurement method^9^.

When fault recording is analyzed by combing time with frequency domain, there is an approach where faults are identified based on a discrete S-transformation and fuzzy decision box according to the obvious differences in the alteration of the time–frequency amplitudes of currents caused by the faults in different distances and types^10^. And auxiliary input signals with a sinusoidal form are designed on the YJBK (Youla, Jabr, Bongiorno, and Kucera) parameterization architecture, which enables the fault detection signal to be transformed from the conventional system output signal to the cumulative sum of the designed residual signals^11^.

When a space vector is applied to fault recording analysis, a method is proposed where the components of fundamental positive sequence, negative sequence, and harmonic components are real-time detected, and three-phase instantaneous values are synthesized into one space vector to make it be synthesized by two rotating vectors in an anticlockwise and clockwise direction and rotated in an appropriate angle, thus various components are separated from the fundamental component in real time and accurately^12,13^.

There are fault detection methods where effective artificial intelligence algorithms are highly applied to fault detection^14^. For example, a novel transformer model based on a deep convolutional neural network (CNN) is proposed, where 1-D deep CNNs are used to extract features in power systems^15^. In a fault detection algorithm based on a random forest (RF), the magnitude of maximum angular difference between positive and zero sequence component of the current at distributed generation buses is computed and then fed to a trained RF classifier for detecting fault conditions^16^. And a data-driven fault detection method with an ensemble classifier is used in modern distribution system^17^.

An extreme learning machine (ELM) algorithm for spontaneous fault detection and fault classification is proposed, which generates ten different types of normal and faulty data by two different transmission lines and normalizes the data to be used as a classifier for training^18^. The fault type detection and localization are achieved according to a histogram-based gradient boost (HGB) algorithm considering hosting capacity amendment in active distribution network^19^. Discrete wavelet transformation (DWT) and back-propagation neural network (BPNN) are also applied to fault diagnosis, where the high-frequency components in fault currents are extracted by DWT and the first-order coefficients detecting faults are studied to construct a BPNN-based decision algorithm^20^.

Besides, Kalman filter^21^, Bayes estimation^22^, petri nets^23^, cluster analysis^24^, support vector machine^25^, fuzzy set theory^26^, and rough set theory^27^ etc. are also widely applied to the area of fault diagnosis in such way as multi-source information fusion. However, a large amount of data in these studies are demanded and the computational processes are cumbersome. This increases the time cost and complexity of fault analysis and limits the depth and breadth of its application in power grids.

It is still not deep enough for all the present strategies of fault analysis to mine the characteristics of electrical physical quantities themselves, and the relevant analyses in terms of the phase domain are not involved. In the algorithm studied in this article artificial intelligent models are not trained, and the research priority is focused on the phase-domain trajectories of fault recording, which is innovated to acquire fault features in a power system.

The research results in the article are listed in “**Results”**, including the expression of the reformulated waveform from its original one, their correlation coefficient, relative error, and the absolute error curves and the maximum absolute errors post both offline and online analysis. The algorithm of calculating fault features in fault recording and reformulating its waveforms, the accuracy requirement and errors are discussed in detail in “**Methods”**. The significance of the research is listed in “**Conclusion”**.

## Results

A current of the fault recording pre and post a three-phase short circuit in an infinite power supply system is shown as follows:

1 $$i\left( t \right) = \left\{ {\begin{array}{*{20}l} {sin\left( {\omega t + 27.6^{^\circ } } \right),} \hfill & {0 \le t < 0.04s} \hfill \\ {4.36sin\left[ {\omega \left( {t - 0.04} \right) - 43.7^{^\circ } } \right] + 3.476e^{{ - (t - 0.04)/0.05}} ,} \hfill & {t \ge 0.04s} \hfill \\ \end{array} } \right.$$

where *ω* is the angular frequency of the current.

A polar coordinate system is created by function “polarplot(theta, rho)” in Matlab, where “theta” and “rho” are polar angle *θ* and polar diameter* ρ*. Substituting *θ* for *ωt* and *ρ* for *i* in Eq. (1), where the system frequency is 50 Hz (*ωt* = 100*π*), there is:

2 $$\rho \left( \theta \right) = \left\{ {\begin{array}{*{20}l} {sin\left( {\theta + 27.6^{^\circ } } \right),} \hfill & {0 \le \theta < 720^{^\circ } } \hfill \\ {4.36sin\left[ {\left( {\theta - 720^{^\circ } } \right) - 43.7^{^\circ } } \right] + 3.476e^{{ - (\theta - 720^{^\circ } )/900^{^\circ } }} ,} \hfill & {\theta \ge 720^{^\circ } } \hfill \\ \end{array} } \right.$$

**Figure 1:**
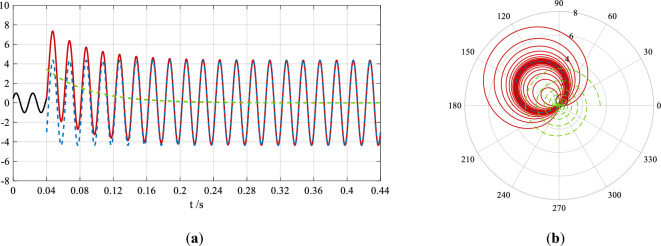
The time-domain waveform and phase-domain trajectory of the current pre and post a three-phase short circuit in an infinite power supply system in the time interval of 0–0.44 s. The colors and styles of the curves in (**a**) and (**b**) are the same. In (**a**) the black solid-line sinusoidal curve is the waveform of the current in the normal steady state pre the short circuit, and the red solid-line curve—the short-circuit current in the transient state, where the blue dash-line sinusoidal and green dash-line exponential curves are the waveforms of its sinusoidal ac component and decaying dc component. In (**b**) the black solid-line circle is the trajectory of the sinusoidal current in the normal steady state, and the red solid-line spiral—the short-circuit current in the transient state, where the blue dash-line circle and green dash-line spiral are the trajectories of its sinusoidal ac component and decaying dc component.

Since *ωt* is the phase angle of current *i*(*t*), and *θ* = *ωt*, Eq. (2) is referred to a phase-domain expression for Eq. (1), which is known as the time-domain expression for the current. Their figures are shown in Fig. 1a,b respectively. The process of forming the trajectory in Fig. 1b from the waveform of the short-circuit current in Fig. 1a is demonstrated by Supplementary Video 1 in Supplementary Information at the end of this article.

Comparing Fig. 1a,b, when the dc component decays, the apple-shaped trajectory becomes smaller and the balloon-shaped trajectory becomes larger, and both of them approach the blue dash-line circle in the short-circuit steady state. These two trajectories are overlapped and stabilize at the position of this circle, as shown by the overlapped red solid-line circle and blue dashed-line circle in Fig. 1b.

The phase-domain trajectory of the dc component in the current is the green dashed-line spiral in Fig. 1b starting from the short-circuit instant and decays around the origin, and one circle is corresponded to one power frequency period. Its polar diameter decays to zero in the short-circuit steady state, and the decaying speed depends on its time constant.

It is challenging to distinguish whether the dc component in the short-circuit current still decays in the waveform at *t* = 0.2 s from Fig. 1a; however, from Fig. 1b at this instant the red solid-line trajectory is still not completely overlapped with the blue dashed-line trajectory, i.e., the current is still in the decaying process.

Due to the complexity of faults themselves and fault recording, it is tough to analyze them both quickly and accurately, and a methodological choice depends on the analysis purpose. When the fault recording is used to find failure causes, determine fault types and identify system operation modes, which are contributed to developing measures to improve the safe and stable operation of a power system, the requirement for real-time analysis is not high, while the requirement for the accuracy of fault feature parameters is high. For this case offline analysis is an approach.

In particular, if there are normal steady states, transient states, short-circuit steady states, and states post the removal of failure in fault recording, periodic and non-periodic components are separated from it by the characteristics of circular and spiral phase-domain trajectories, and other components such as harmonics are considered further.

Fault recording is also included in microcomputer relay protection and automation, and it is mainly for the purpose of identifying fault types and determining fault distances in order to decide whether to send a tripping signal to a tripping circuit. In this case the real-time requirement for fault recording analysis is relatively high, while the requirement for the accuracy of fault feature parameters is not as high as that in offline analysis. Therefore, online analysis is suitable in this case.

Whether offline or online analysis is applied to fault recording, there inevitably are errors between a reformulated time-domain waveform and its original one. Therefore, only the reformulated waveform which meets certain accuracy requirement is the analysis result.

In the offline analysis of the short-circuit current in Fig. 1a, a correlation coefficient (*c*_*xy*_) or relative error (*ε*) is used to reduce the absolute errors (*e*) in the reformulated waveform. When the accuracy requirement is that the correlation coefficient between the reformulated waveform and its original one is greater than or equal to *c*_*M*_ = 1–10^−^^10^ (0.999 999 999 999 9), the following reformulated waveform is obtained:

3 $$i\prime (t) = \left\{ {\begin{array}{*{20}l} {sin\left( {100\pi t + 27.59941406^{^\circ } } \right),} \hfill & {0 \le t < 0.04s} \hfill \\ \begin{gathered} 4.36sin\left[ {100\pi \left( {t - 0.04} \right) - 43.69941406^{^\circ } } \right] \hfill \\ \quad + 3.475967764e^{{ - (t - 0.044)/0.0500003072262776}} , \hfill \\ \end{gathered} \hfill & {t \ge 0.04s} \hfill \\ \end{array} } \right.$$

Between the waveforms in Eqs. (3) and (1) the correlation coefficient, relative error, and maximum absolute error are:

$$\begin{gathered} c_{xy} = 0.{999 999 999 999 951 432},\varepsilon = {4}.{856 781 643 525 21} \times {1}0^{{ - {11}}} , \hfill \\ e_{{{\text{max}}}} = {6}.{393} \times {1}0^{{ - {5}}} . \hfill \\ \end{gathered}$$

**Figure 2:**
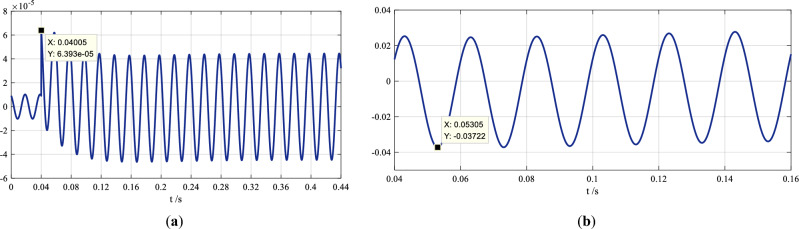
The absolute error curves of the reformulated waveforms of the fault recording. The figure in (**a**) is the errors in the reformulated waveform in offline analysis, and the figure in (**b**)—in online analysis. The maximum absolute errors are all marked in the two figures.

The curve of the absolute errors is shown in Fig. 2a.

In the online analysis only the waveform of the first 6 periods in Fig. 1a is used because the action time of regular quick protection is up to 0.12s^28^. When calculating the fault feature parameters, there is no accuracy requirement for the reformulated waveform since the rapidity is the primary consideration in protective relaying.

The online analysis of the sinusoidal current in the normal steady state and the adjustment process of its reformulated waveform are completely the same as that in offline analysis. The reformulated time-domain waveform of the short-circuit current in the transient state is:

4 $${i}_{k}{\prime}\left(t\right)=4.354sin\left[100\pi \left(t-0.04\right)-44.1^\circ \right]+{3.481 507 286 669 61e}^{-(t-0.04)/0.050 141 175 233 319 }, t\ge 0.04s$$

Between the waveforms in Eqs. (4) and (1) the correlation coefficient and relative error are:

$$c_{xy} = 0.{999 980 063 830 419},\varepsilon = {3}.{987 194 171 128 92} \times {1}0^{{ - {5}}} .$$

The maximum absolute error in the first two periods (40 ms) is *e*_max_ =  − 0.03722. The curve of the absolute errors in the time interval of 0.04–0.16 s is shown in Fig. 2b.

## Methods

### The relationship between the time-domain waveform of the current and its phase-domain trajectory

**Figure 3:**
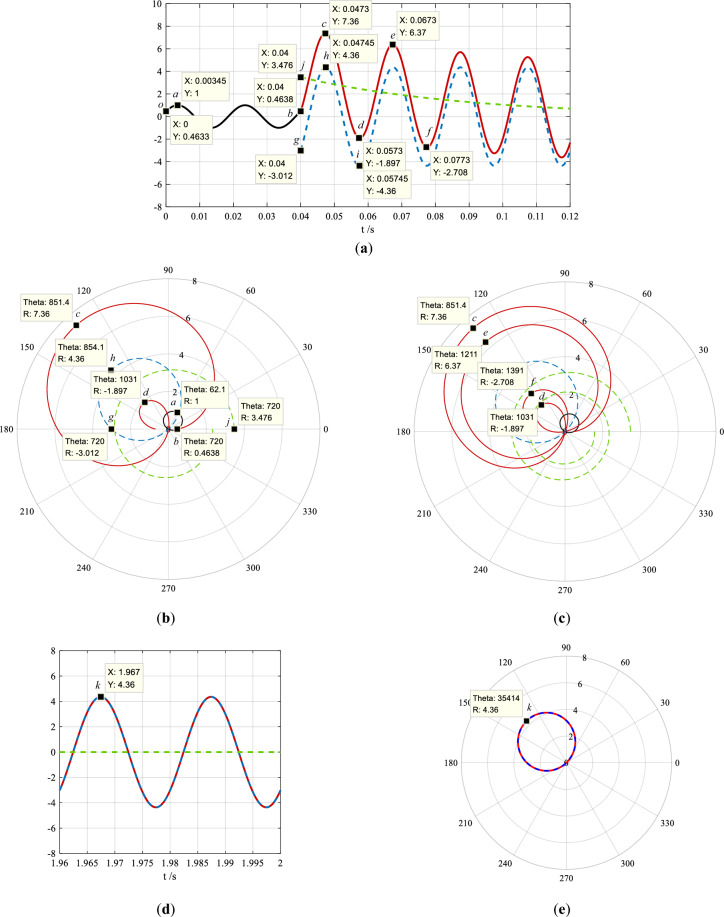
The time-domain waveforms of the current expressed by Eq. (1) and its phase-domain trajectories expressed by Eq. (2). The waveform in the time interval of 0–0.12 s is shown in (**a**), its trajectories in the time interval of 0–0.06 s and 0–0.08 s are shown in (**b**) and (**c**), the waveform and its trajectory in the time interval of 1.96–2 s are shown in (**d**) and (**e**). The black solid lines indicate the current in the normal steady state, and the red solid lines—in the transient state. The blue dash lines indicate the sinusoidal ac component, and the green dash lines—the decaying dc component.

The time-domain waveforms of the current in Eq. (1) and its phase-domain trajectories in Eq. (2) in the time interval of 0–0.12 s, 0–0.06 s, 0–0.08 s, and 1.96–2 s are shown in Fig. 3a–e.

The fault recording of the current is digitally sampled from the instant when its phase angle is equal to 27.6°, and this instant is taken as the 0-th sample point, which is also described as a reference point, and there are: *t* = 0 s and *θ* = 0°. The phase angle of the reference point is equal to the initial phase angle of the current, which is *φ*_0_ = 27.6°.

The coordinates of the points (*a*–*k*) in the waveforms shown in Fig. 3a,d are corresponded with those in the trajectories shown in Fig. 3b,c,e, which are listed in Table 1 respectively.

**Table 1:** The comparison of the coordinates of the points (*a*–*k*) in the waveforms (shown in Fig. 3a,d) with those in trajectories (shown in Fig. 3b,c,e). In the table “Theta” expressed by “angle 1 / angle 2” is the polar angle, where “angle 1” is the phase angle of a point based on the reference point, “angle 2” is the value which “angle 1” is converted to between 0° and 360°. “Position *θ*_*m*_” is the polar angle between 0° and 360°, and when the polar diameter of a point is negative, *θ*_*m*_ is the polar angle of the positive polar diameter to which the point is reflected through the origin.

Point	Time-domain waveform *i*(*t*)		Phase-domain trajectory *ρ*(*θ*)		
	X: *t*(s)	Y: *i*	Theta: *θ*(°)	R: *ρ*	Position *θ*_*m*_(°)
*o*	0	0.463 3	0	0.463 8	0
*a*	0.003 45	1	62.1 / 62.1	1	62.1
*b*	0.04	0.463 8	720 / 0	0.463 8	0
*c*	0.047 3	7.36	851.4 / 131.4	7.36	131.4
*d*	0.057 55	− 1.897	1031 / 311	− 1.897	131
*e*	0.067 3	6.37	121 1 / 131	6.37	131
*f*	0.077 3	− 2.708	1391 / 311	− 2.708	131
*g*	0.04	− 3.012	720 / 360	− 3.012	180
*h*	0.047 45	4.36	854.1 / 134.1	4.36	134.1
*i*	0.057 45	− 4.36	1034.1 / 314.1	− 4.36	134.1
*j*	0.04	3.476	720 / 0	3.476	0
*k*	1.967	4.36	354 14 / 134	4.36	134

Point *o*: the reference point, initial phase angle of the current in the normal steady state, *φ*_0_ = 27.6°;

Point *a*: the 1st crest instant of the current in the normal steady state in the waveforms, the position of the 1st black circle in the trajectories;

Point *b*: the short-circuit instant in the waveforms, the start of the maximum apple-shaped curve, i.e., the short-circuit reference point in the trajectories;

Point *c*: the 1st crest instant of the short-circuit current in the waveforms, the position of the maximum polar diameter of the maximum apple-shaped curve in the trajectories;

Point *d*: the 1st trough instant of the short-circuit current in the waveforms, the position of the maximum polar diameter of the minimum balloon-shaped curve in the trajectories;

point *e*: the 2nd crest instant of the short-circuit current in the waveforms, the position of the maximum polar diameter of the 2nd largest apple-shaped curve in the trajectories;

Point *f*: the 2nd trough instant of the short-circuit current in the waveforms, the position of the maximum polar diameter of the 2nd smallest balloon shaped curve in the trajectories;

Point *g*: the initial instant of the short-circuit current in the short-circuit steady state in the waveforms, the start of the blue dashed-line circle in the trajectories;

Point *h*: the 1st crest instant of the short-circuit current in the short-circuit steady state in the waveforms, the position of the diameter of the blue dashed-line circle in the trajectories, which is through the origin;

Point *i*: the 1st trough instant of the short-circuit current in the short-circuit steady state in the waveforms, it is overlapped with point *h* in the trajectories;

Point *j*: the initial instant of the decaying dc component in the waveforms, the start terminal of the green dashed-line spiral in the trajectories;

Point *k*: one of the crest instants of the short-circuit current in the short-circuit steady state in the waveforms when the dc component decays to end, the position of the diameter of the red solid-line circle in the trajectories, which is through the origin.

In Fig. 3b the black solid-line circle containing point *a* and *b* is the trajectory of the sinusoidal current in the normal steady state. Point *b* is the short-circuit instant (the 0-th sample point or the reference point), and also the start of the apple-shaped curve. Since the decaying dc component is greater than zero, the 1st crest located above the time axis is corresponded to the position of the maximum polar diameter of the maximum apple-shaped curve, and the 1st trough located below the time axis—the minimum balloon-shaped curve. When the dc component decays, the “apple” becomes gradually smaller and the “balloon”—larger, and both of them approach the circle of the sinusoidal current in the short-circuit steady state and stabilize at the position of this circle, as shown by the overlapped red solid-line circle and blue dashed-line circle in Fig. 3e. The 1st largest apple and the 1st smallest balloon are shown in Fig. 3b, and the 2nd larger apple and the 2nd smaller balloon—in Fig. 3c.

The phase-domain trajectory of the sinusoidal ac component in the current in the short-circuit steady state is a circle too, which starts from the short-circuit instant (point *g*) and is also the trajectory of the sinusoidal current in the short-circuit steady state, i.e., the blue dashed-line circles shown in Fig. 3b,c,e.

The phase-domain trajectory of the decaying dc component in the current is the spiral, which is the green dashed-line spiral in Fig. 3b,c. It starts from the short-circuit instant (point *j*) and decays around the origin, and rotates one circle per power frequency period. Its polar diameter decays to zero in the short-circuit steady state, and its decaying speed depends on the time constant of the exponential function.

After the phase-domain trajectory of a short-circuit current (Eq. (1)) is formed by an appropriate way, which is the expression in Eq. (2), the following short-circuit fault feature parameters are accessible from analyzing the trajectory with its time-domain waveform: the amplitude and initial phase angle of the sinusoidal current in the normal steady state, the short-circuit initial phase angle (the initial phase angle of the sinusoidal ac component at the short-circuit instant), the value of the current at the short-circuit instant, the initial value and time constant of the decaying dc component, the amplitude of the sinusoidal current in the short-circuit steady state. The original waveform of the current is thus reformulated by these features.

### The offline analysis of fault recording

#### Calculating the time-domain waveform from the circular phase-domain trajectory of the sinusoidal current in the normal steady state

The phase-domain trajectory of a sinusoid is circular, and the diameter of the circle is equal to its amplitude. The relationship between the position (*θ*_*m*_) of the circle (the polar angle of the diameter through the origin) and the initial phase angle (*φ*_0_) of the sinusoid is:

5 $${\varphi }_{0}+{\theta }_{m}=90^\circ$$

It is uncomplicated to obtain the amplitude and initial phase angle of the sinusoid from the diameter of the circular trajectory and its position. An especial attention should be paid to that when the polar diameter of this diameter is positive, it is more convenient to convert its polar angle to the value between 0° and 360°. If the polar diameter is negative, it should be firstly reflected through the origin to the position where its polar diameter is positive, and then its polar angle is converted to the convenient value between 0° and 360°.

For instance, in the trajectory (Fig. 3b) of the sinusoidal current in the normal steady state, point *a* is the position of the diameter (through the origin) of the trajectory circle, its polar diameter and polar angle are 1 and 62.1° respectively, which means that the position of the circle is *θ*_*m*_ = 62.1°. Therefore, the amplitude of the sinusoid is 1 and its calculated initial phase angle is obtained by Eq. (5), which is:

$${\varphi }_{0C}= 90^\circ -62.1^\circ =27.9^\circ$$

The polar angle and polar diameter of point* o* (the reference point) and *b* (the short-circuit reference point) are found from the trajectory circle in Fig. 3b, which is also list in Table 1. Supposing the duration of the sinusoidal current in the normal steady state is 0.04 s, and its frequency is 50 Hz, the expression of the raw reformulated waveform of this current is:

6 $${i}_{|0|}\left(t\right)=sin\left(100\pi t+27.9^\circ \right), 0\le t<0.04s$$

**Figure 4:**
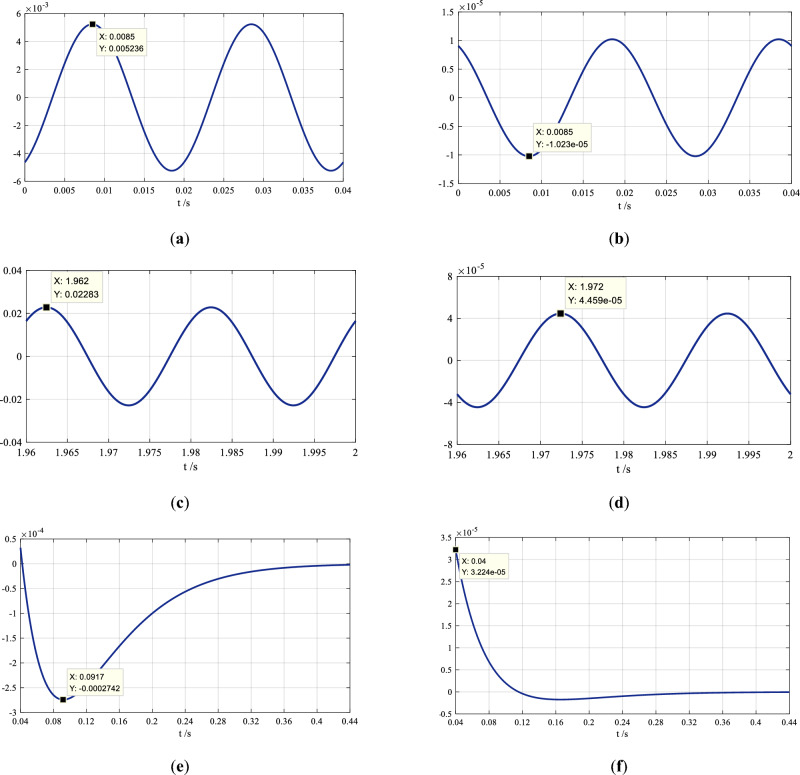
The figures in (**a**) and (**b**) are the absolute error curves of the reformulated waveforms of the sinusoidal current in the normal steady state pre and post the adjustment. The figures in (**c**) and (**d**) are the absolute error curves of the reformulated waveforms of the sinusoidal current in the short-circuit steady state pre and post the adjustment. The figures in (**e**) and (**f**) are the absolute error curves of the reformulated waveforms of the decaying dc component in the short-circuit current.

Comparing Eq. (6) with the original function in Eq. (1), the calculated initial phase angle (*φ*_0*C*_ = 27.9°) from the trajectory circle is not equal to the actual angle (*φ*_0_ = 27.6°), and this results in errors between the raw reformulated waveform and the original one. The absolute error curve is shown in Fig. 4a, where the maximum absolute error (*e*_max_ = 0.005 236) is marked.

#### The correlation analysis between the reformulated waveform and its original one in the normal steady state

After the features such as the amplitude and initial phase angle of the sinusoid from the circular trajectory are obtained, the similarity between the reformulated waveform and the original one is required to be measured by certain indexes. Obviously, it is not appropriate to use the absolute error curve in Fig. 4a.

The similarity or interdependency between the two signals of *x*(*t*) and *y*(*t*) is measured by a correlation coefficient (*c*_*xy*_) or a relative error (*ε*). When *y*(*t*) is used to approximate to *x*(*t*) in the time interval of *t*_1_–*t*_2_, the correlation coefficient *c*_*xy*_ is calculated by the following equation^29^:

7 $${c}_{xy}=\underset{{t}_{1}}{\overset{{t}_{2}}{\int }}x\left(t\right)y\left(t\right)dt/\left(\sqrt{{\int }_{{t}_{1}}^{{t}_{2}}{x}^{2}(t)dt}\sqrt{{\int }_{{t}_{1}}^{{t}_{2}}{y}^{2}(t)dt}\right)$$

And the relative error *ε* is:

8 $$\varepsilon =1-{c}_{xy}^{2}$$

The actual initial angle (*φ*_0_) of the original sinusoidal waveform is unknown pre its determination. In order to obtain the calculated initial angle (*φ*_0*C*_) within the allowable error range, a sample window (Δ*φ*) (its definition and calculation are found below) is taken as an adjustment range of the phase angle, and *φ*_0_ is located in the interval which is centered on *φ*_0*C*_:

$${[\varphi }_{0C}-\Delta \varphi , {\varphi }_{0C}+\Delta \varphi ]$$

Taking this interval as an initial one and *φ*_0*C*_ as an initial value, the initial angle which meets the requirement for an accuracy will be found in the interval by a dichotomy method. The requirement is expressed by the maximum correlation coefficient (*c*_*M*_) between the reformulated waveform and its original one, and when the coefficient calculated by Eq. (7) meets the condition of *c*_*xy*_ ≥ *c*_*M*_, the adjustment process is ended. The middle value of the present interval is exactly the calculation result of the initial angle.

**Figure 5:**
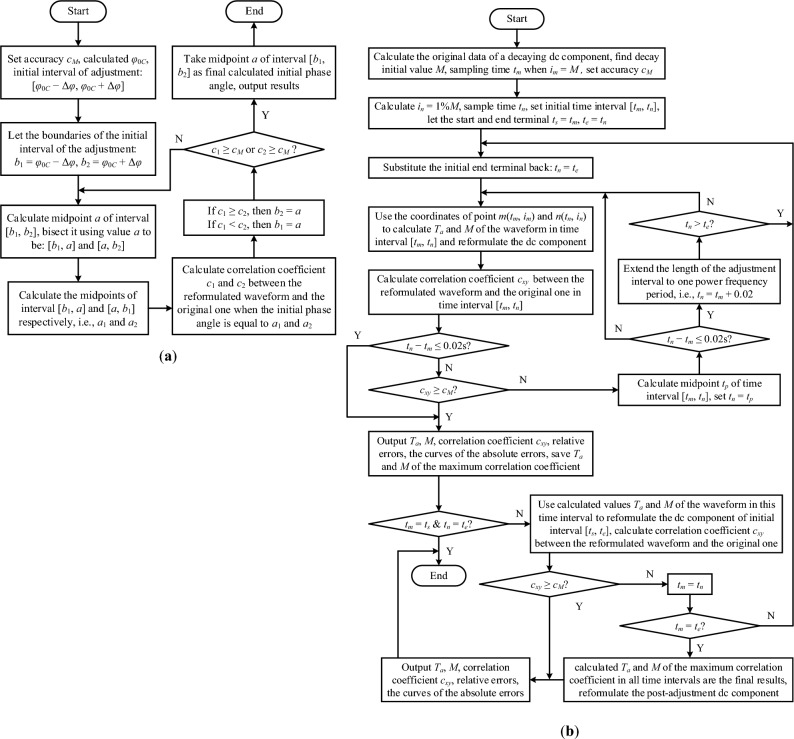
The block chart for the adjustment algorithm of the initial phase angle of a sinusoidal current is (**a**), and (**b**) is the block chart of the adjustment algorithm of the decaying dc component in a short-circuit current.

The calculated initial phase angle from the trajectory circle in Fig. 3b is *φ*_0*C*_ = 27.9°, and it is the initial value. Taking 0.9° (the value of the sample window) as the adjustment range, the actual initial phase angle (27.6°) is located in the interval of [27.0°, 28.8°]. The adjustment algorithm is shown in Fig. 5a, the accuracy requirement is that the correlation coefficient *c*_*xy*_ is not less than *c*_*M*_ = 1–10^−10^ (0.999 999 999 9), and the data in the adjustment process are listed in Table 2.

The program list of adjusting process of the sinusoidal current in the normal steady state, which is corresponding to the block chart in Fig. 5a, is referred to Supplementary Information 1 in Supplementary Information at the end of this article.

**Table 2:** The data in the adjustment process of the initial phase angle of the sinusoidal waveform in the normal steady state, where the relative errors (*ε*) are calculated by Eq. (8), the maximum absolute errors (*e*_max_) are the maximum among the absolute errors between the reformulated waveform and its original one.

*n*	*b*_1_(°)	*b*_2_(°)	*φ*_0*C*_ (°)	(*φ*_0*C*_–*φ*_0_) (°)	*c*_*xy*_	*ε*	*e*_max_
0	27	28.8	27.9	0.3	0.999986292247427	2.74153 × 10^−5^	0.005235964
1	27	27.9	27.45	− 0.15	0.999996573055985	6.85388 × 10^−6^	− 0.002617937
2	27.45	27.9	27.675	0.075	0.999999143263629	1.71347 × 10^−6^	0.001308983
3	27.45	27.675	27.5625	− 0.0375	0.999999785815884	4.28368 × 10^−7^	0.000654488
4	27.562 5	27.675	27.61875	0.01875	0.999999946453970	1.07092 × 10^−7^	0.000327245
5	27.562 5	27.61875	27.590625	− 0.009375	0.999999986613492	2.6773 × 10^−8^	− 0.000163622
6	27.590 625	27.61875	27.6046875	0.0046875	0.999999996653373	6.69325 × 10^−9^	− 8.18112 × 10^−5^
7	27.590 625	27.6046875	27.59765625	− 0.00234375	0.999999999163343	1.67331 × 10^−9^	− 4.09056 × 10^−5^
8	27.59765625	27.6046875	27.60117188	0.001171875	0.999999999790836	4.18328 × 10^−10^	2.04528 × 10^−5^
9	27.59765625	27.60117188	27.59941406	− 0.000585938	0.999999999947709	1.04582 × 10^−10^	− 1.02264 × 10^−5^

When *n* = 9, *c*_*xy*_ has exceeded *c*_*M*_ and the adjustment result is *φ*_0*C*_ = 27.599 414 06°, which is smaller than the actual one (27.6°) by 0.000 585 938°. The reformulated sinusoidal function meeting the accuracy requirement is changed from Eq. (6) to the following one:

9 $$i_{{|0|}}^{\prime } (t) =sin\left(100\pi t+27.599 414 06^\circ \right), 0\le t<0.04s$$

The curve of the absolute errors between the reformulated sinusoidal waveform and its original one is shown in Fig. 4b, where the maximum absolute error *e*_max_ =  − 1.023 × 10^−5^ is marked.

#### Calculation of the sinusoidal waveform from the circular trajectory of the sinusoidal current in the short-circuit steady state and the correlation analysis between the reformulated waveform and its original one

Following the same method as analyzing the sinusoidal current in the normal steady state, the amplitude and the calculated short-circuit initial phase angle of the sinusoidal waveform (Fig. 3d) in the time interval 1.96–2 s in the short-circuit steady state are obtained from the polar diameter of 4.36 and the polar angle of 354 14° (or 134°) of point *k* in the trajectory in Fig. 3e (or Table 1), and they are 4.36 and *φ*_0*kC*_ =  − 44° respectively. Taking point *o* as the reference point, the expression of the raw reformulated function of the waveform in this time interval is:

10 $${i}_{p}(t)=4.36sin\left[100\pi \left(t-1.96\right)-44^\circ \right], 1.96s\le t<2s$$

There is an error between the calculated initial phase angle (*φ*_0*kC*_ =  − 44°) and the actual one (*φ*_0*k*_ =  − 43.7°). The curve of the absolute errors between the initial reformulated waveform and the original one caused by the error is shown in Fig. 4c, where the maximum absolute error *e*_max_ =  − 0.022 83 is marked.

Taking − 44° as the initial value and 0.9° as the adjustment range, the actual angle of − 43.7° is located in the initial interval of [− 44.9°, − 43.1°]. Following the adjustment algorithm in Fig. 5a, the accuracy requirement is taken as the same one as in the normal steady state (the correlation coefficient is not less than *c*_*M*_ = 1–10^−10^), the data in the adjustment process are listed in Table 3.

The program list of adjusting process of the sinusoidal ac component in the short-circuit current, which iscorresponding to the block chart in Fig. 5, is referred to Supplementary Information 1 in Supplementary Information at the end of this article.

**Table 3:** The data in the adjustment process of the short-circuit initial phase angle of the sinusoidal waveform in the short-circuit steady state.

*n*	*b*_1_(°)	*b*_2_(°)	*φ*_0*C*_ (°)	(*φ*_0*C*_ − *φ*_0_) (°)	*c*_*xy*_	*ε*	*e*_max_
0	− 44.9	− 43.1	− 44	− 0.3	0.999986292247427	2.74153 × 10^−5^	0.022828663
1	− 44	− 43.1	− 43.55	0.15	0.999996573055985	6.85388 × 10^−6^	0.011414136
2	− 44	− 43.55	− 43.775	− 0.075	0.999999143263629	1.71347 × 10^−6^	0.005707112
3	− 43.775	− 43.55	− 43.6625	0.0375	0.999999785815885	4.28368 × 10^−7^	0.002853537
4	− 43.775	− 43.6625	− 43.71875	− 0.01875	0.999999946453970	1.07092 × 10^−7^	0.001426773
5	− 43.71875	− 43.6625	− 43.690625	0.009375	0.999999986613492	2.6773 × 10^−8^	0.000713386
6	− 43.71875	− 43.690625	− 43.7046875	− 0.0046875	0.999999996653373	6.69325 × 10^−9^	0.000356693
7	− 43.7046875	− 43.690625	− 43.69765625	0.00234375	0.999999999163343	1.67331 × 10^−9^	0.000178346
8	− 43.7046875	− 43.69765625	− 43.70117188	− 0.001171875	0.999999999790836	4.18328 × 10^−10^	8.91732 × 10^−5^
9	− 43.70117188	− 43.69765625	− 43.69941406	0.000585938	0.999999999947709	1.04582 × 10^−10^	4.45866 × 10^−5^

When *n* = 9, the correlation coefficient between the reformulated function and its original one has exceeded *c*_*M*_. The adjustment result is *φ*_0*kC*_ =  − 43.699 414 06° and is larger than actual angle (− 43.7°) by 0.000 585 938°. The reformulated sinusoidal function meeting the accuracy requirement is changed from Eq. (10) to the following one:

11 $${i}_{p}{\prime}(t)=4.36sin\left[100\pi \left(t-1.96\right)-43.699 414 06^\circ \right], 1.96s\le t<2s$$

The curve of the absolute errors between the reformulated sinusoidal waveform and the original one is shown in Fig. 4d, where the maximum absolute error *e*_max_ = 4.459 × 10^−5^ is marked.

#### Calculation of the decaying dc component from the time-domain waveform of the short-circuit current and the correlation analysis between the reformulated waveform and its original one

Equation (11) is the sinusoidal current in the short-circuit steady state, from it the expression of the sinusoidal ac component in the time interval of 0.04–0.44 s in the transient process is directly written to be:

12 $$i_{p}^{\prime } (t) =4.36sin\left[100\pi \left(t-0.04\right)-43.699 414 06^\circ \right], 0.04s\le t<0.44s$$

Subtracting the sinusoidal ac component (Eq. (12)) from the short-circuit current (Eq. (1)), the raw expression of the decaying dc component is:

13 $${i}_{ap}(t)=3.476{e}^{-(t-0.04)/0.05}-1.022 658 222\times {10}^{-5}cos[100\pi (t-0.04)-43.699 707 03^\circ ], 0.04s\le t<0.44s$$

**Figure 6:**
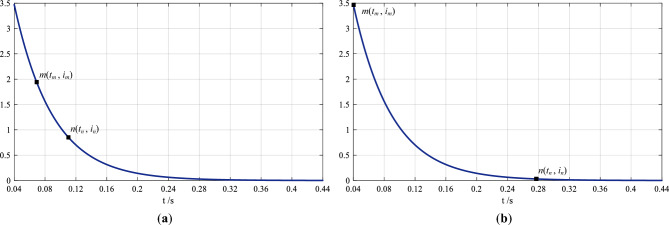
The waveform of the raw expression of the decaying dc component in the short-circuit current and the calculation of its time constant (*T*_*a*_) and initial value (*M*). In (**a**) point *m* and *n* are any two points on the waveform. In (**b**) point *m* is the one where *i*_*m*_ = *M*, and point *n* is the one where *i*_*n*_ ≤ 1%*M*.

The waveform is plotted in Fig. 6a,b.

The initial phase angle is − 43.699 414 06° from the reformulated sinusoidal ac component (see Eq. (12)), and the actual one is − 43.7° (see Eq. (1)). The difference causes the errors in the expression in Eq. (13), and the waveform in Fig. 6a,b is not accurate dc component. The errors are:

$${\Delta i}_{ap}\left(t\right)=-1.022 658 222\times {10}^{-5}cos[100\pi (t-0.04)-43.699 707 03^\circ ], 0.04s\le t<0.44s$$

Such small errors cannot be obviously observed in Fig. 6a,b.

Expressing the initial value and time constant of the dc component as *M* and *T*_*a*_, its expression of the waveform is:

14 $${i}_{ap}\left(t\right)=M{e}^{-(t-0.04)/{T}_{a}},0.04s\le t<0.44s$$

Taking any two points in the waveform: point *m*(*t*_*m*_, *i*_*m*_) and point *n*(*t*_*n*_, *i*_*n*_) (see Fig. 6a), and substituting their coordinates into Eq. (14), there are:

$$\left\{\begin{array}{c}{i}_{m}=M{e}^{-({t}_{m}-0.04)/{T}_{a}}\\ {i}_{n}=M{e}^{-({t}_{n}-0.04)/{T}_{a}}\end{array}\right.$$

Solving this set, the time constant (*T*_*a*_) and initial value (*M*) are obtained as follows:

15 $$\left\{\begin{array}{c}{T}_{a}=\left({t}_{n}-{t}_{m}\right)/(ln{i}_{m}-{lni}_{n})\\ M={i}_{m}{e}^{({i}_{m}-0.04)/{T}_{a}}\end{array}\right.$$

Substituting *T*_*a*_ and *M* back into Eq. (14), the expression of the raw reformulated waveform of the dc component is then obtained, and there inevitable are errors in it. Taking the waveform of a segment from the time intervals of the waveform as an adjustment object, calculating its time constant (*T*_*a*_) and initial value (*M*) by Eq. (15) using the coordinates of the two boundary points, and an expression of the dc component is formed. When the accuracy requirement between the reformulated waveform and the original one (see Eq. (13)) is met, the adjustment process is completed.

Taking the initial instant where the dc component starts to decay as a start terminal (point *m*(*t*_*m*_, *i*_*m*_)) and the instant where it decays to just less than or equal to 1% of the initial value (*M*)—an end one (point *n*(*t*_*n*_, *i*_*n*_)), the waveform of this segment is described as an initial adjustment object, and the duration from the start terminal to the end one—an initial time interval [*t*_*m*_, *t*_*n*_], as shown in Fig. 6b, there are:

$${i}_{m}=M, {i}_{n}\le 1\%M$$

The value of correlation coefficient *c*_*M*_ between the reformulated waveform and the original one is still taken as an accuracy requirement. The time constant (*T*_*a*_) and decaying initial value (*M*) of the initial adjustment object are calculated by Eq. (15) using the coordinates of the two boundary points (*m*(*t*_*m*_, *i*_*m*_) and *n*(*t*_*n*_, *i*_*n*_)), and they are substituted into Eq. (14) to calculate correlation coefficient *c*_*xy*_ between the reformulated waveform and the original one. When the condition of *c*_*xy*_ ≥ *c*_*M*_ is satisfied, the reformulated dc component meets the accuracy requirement, and the adjustment process of the waveform is completed. When the condition of *c*_*xy*_ ≥ *c*_*M*_ is not satisfied, the waveform of the initial adjustment object is dichotomized into two segments, the first one is taken as a new adjustment object. After obtaining its *T*_*a*_ and *M* by Eq. (15), another waveform of the dc component is reformulated to calculate the correlation coefficient to determine whether its accuracy meets the requirement.

The adjustment process is detailed in the block chart shown in Fig. 5b, where after *T*_*a*_ and *M* of each segment are calculated, whether the accuracy of the reformulated waveform meets the requirement, the initial adjustment object is reformulated using these *T*_*a*_ and *M*. If the accuracy of the reformulated waveform meets the requirement, there is no need to continue the subsequent adjustment process, this reformulated waveform is the final result.

In the adjustment process of the dc component, if the duration of any segment is equal to one power frequency period, regardless of the accuracy of the reformulated waveform, there is no need to continue the subsequent adjustment in this time interval. After the adjustment process is completed, if all time intervals are equal to or less than one power frequency period and all the reformulated waveforms do not meet the accuracy requirement, the values of *T*_*a*_ and *M* of the segment with the largest correlation coefficient are taken to reformulate the dc component, and it is also the final result of the adjustment.

The program list of adjusting process of the decaying dc component in the short-circuit current, which is corresponding to the block chart in Figure 5b, is referred to Supplementary Information 2 in Supplementary Information at the end of this article.

In the waveform of the dc component in Fig. 6b, the short-circuit instant is taken as point *m*, which is:

$${t}_{m}=0.04{\text{s}},{i}_{m}=3.475 967 764$$

The value of 3.475 967 764 is actually the initial value of the dc component, which is *M* = 3.475 967 764. When the dc component decays to less than 1% of the initial value (*M*), the decaying process is nearly completed, therefore, the point where the dc component is less than or equal to 1%*M* is taken as point *n*, which is:

$${t}_{n}=0.270 35{\text{s}},{i}_{n}=0.034 7$$

The time constant and initial value are calculated by Eq. (15) using the two coordinates (*t*_*m*_, *i*_*m*_) and (*t*_*n*_, *i*_*n*_) to be:

$${T}_{a}=0.050 011 177 313 940 1{\text{s}}, M=3.475 967 764$$

Substituting them into Eq. (14), the expression of the reformulated dc component is:

16 $${i}_{ap}\left(t\right)=3.475 967 764{e}^{-(t-0.04)/0.050 011 177 313 940 1},0.04s\le t<0.44s$$

Taking the time interval of 0.04–0.270 35 s as the initial time interval of the adjustment, the data in the adjustment process are listed in Table 4, where the accuracy requirement is: the correlation coefficient (*c*_*xy*_) is not less than *c*_*M*_ = 1–10^−10^ (0.999 999 999 9). The time constant and decay initial value of the dc component meeting the accuracy requirement are:

**Table 4:** The data in the adjustment process of the decaying dc component in the short-circuit current.

*n*	*t*_*m*_(s)	*t*_*n*_(s)	*M*	*T*_*a*_ (s)	Δ*T*_*a*_ (s)	*c*_*xy*_	*ε*	*e*_max_
0	0.04	0.27035	3.475967764	0.0500111773139401	1.11773 × 10^−5^	0.999999993807655	1.23847 × 10^−8^	− 0.00027421
1	0.04	0.0688	3.475967764	0.0500003072262776	3.07226 × 10^−7^	0.999999999998369	3.26295 × 10^−12^	3.22357 × 10^−5^

$${T}_{a}=0.050 000 307 226 277 64{\text{s}}, M=3.475 967 764$$

The expression of the reformulated decaying dc component is:

17 $$i_{{ap}}^{\prime } (t) = 3.475967764e^{{ - (t - 0.04)/0.05000030722627764}},0.04s\le t<0.44s$$

Equations (16) and (17) are the expressions of the dc component pre- and post-adjustment, and the curves of the absolute errors between either of them and the expression in Eq. (13) are shown in Fig. 4e,f, where the maximum absolute errors are also marked.

### The online analysis of fault recording

The online analysis of the sinusoidal current in the normal steady state and the adjustment process of the reformulated waveform are completely the same as that in offline analysis. Due to the lack of the sinusoidal current in the short-circuit steady state in quick protection, it is trickier to separate the sinusoidal ac component from the short circuit current before analyzing the dc component than the above-mentioned offline analysis.

#### Estimating the decaying dc component and sinusoidal ac component in the short circuit current in 40 ms

The regular expression of the short-circuit current in Fig. 3a is:

18 $${i}_{k}\left(t\right)={i}_{p}\left(t\right)+{i}_{ap}\left(t\right)={A}_{k}\mathit{sin}\left[100\pi \left(t-0.04\right)+{\varphi }_{0k}\right]+M{e}^{-(t-0.04)/{T}_{a}}, 0.04s\le t\le 0.12s$$

The decrease of the “apple trajectory” and increase of the “balloon trajectory” as well as their polar angles are utilized to calculate the decaying dc component in the current, as shown in Fig. 3c. Point *c* and *e* are the positions of the maximum polar diameters of the largest and second largest apples, and point *d* and *f* are the positions of the maximum polar diameters of the smallest and second smallest balloons. The corresponded to them same points are also shown in Fig. 3a, and the polar diameters and polar angles of these points are listed in Table 1. Substituting the coordinates of these points into Eq. (18), there are:

$$\left\{\begin{array}{c}{A}_{k}{\text{sin}}\left[100\pi \left(0.0473-0.04\right)+{\varphi }_{0k}\right]+M{e}^{-(0.0473-0.04)/{T}_{a}}=7.36 \\ {A}_{k}{\text{sin}}\left[100\pi \left(0.0673-0.04\right)+{\varphi }_{0k}\right]+M{e}^{-(0.0673-0.04)/{T}_{a}}=6.37 \\ {A}_{k}{\text{sin}}\left[100\pi \left(0.0573-0.04\right)+{\varphi }_{0k}\right]+M{e}^{-(0.0573-0.04)/{T}_{a}}=-1.897\\ {A}_{k}{\text{sin}}\left[100\pi \left(0.0773-0.04\right)+{\varphi }_{0k}\right]+M{e}^{-(0.0773-0.04)/{T}_{a}}=-2.708\end{array}\right.$$

Since the time interval between point *c* and *e* and that between point *d* and *f* are all one power frequency period, the sinusoidal ac components in the first two equations are equal, and the ones in the second two equations are also equal. The above equations are simplified to be:

19 $$\left\{\begin{array}{c}M\left({e}^{-0.0073/{T}_{a}}{-e}^{-0.0273/{T}_{a}}\right)=0.99 \\ M\left({e}^{-0.0173/{T}_{a}}{-e}^{-0.0373/{T}_{a}}\right)=0.811\end{array}\right.$$

Solving Eq. (19), there are:

$${T}_{a}=0.050 141 175 233 319 0{\text{s}}, M=3.481 507 286 669 61$$

Substituting the two values of *T*_*a*_ and *M* into Eq. (18), the expression of the decaying dc component is:

20 $$i_{{ap}}^{\prime } (t) = 3.48150728666961e^{{ - (t - 0.04)/0.050141175233319}} ,0.04s \le t \le 0.08s$$

**Figure 7:**
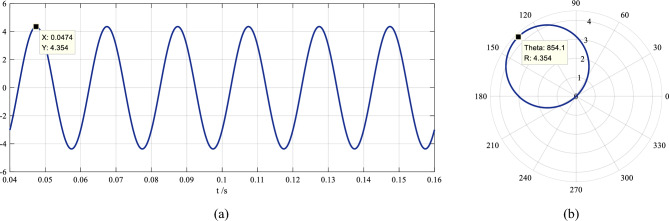
The sinusoidal ac component obtained after the decaying dc component is subtracted from the short-circuit current, where (**a**) is the time-domain waveform of 6 periods post the short circuit and (**b**) is the phase-domain trajectory of the first half period of the waveform in (**a**).

Subtracting this dc component from the short-circuit current (see Eq. (18)), the waveform of the sinusoidal ac component is obtained and shown in Fig. 7a, where there are only 6 periods post the short circuit. Taking the instant at *t* = 0 as a reference point, the phase-domain trajectory of the first half period (0.04–0.05 s) of the waveform is plotted in Fig. 7b.

Following the same way as that in the offline analysis, from the polar angle of 854.1° (or 134.1°) and the polar diameter of 4.354 of the trajectory circle shown in Fig. 7b, the expression of the sinusoidal ac component is immediately obtained:

21 $$i_{p}^{\prime } (t) = 4.354sin[100\pi \left( {t - 0.04} \right) - 44.1^{^\circ } ],0.04s \le t \le 0.08s$$

Since the dc component is calculated by two periods (0.04–0.08 s), at least 40 ms are needed to obtain the decaying dc component and the sinusoidal ac component of the current in this kind of online analysis.

#### Estimating the sinusoidal ac component in the short circuit current in 20 ms

The decaying dc component in the short-circuit current impacts on only the current values but not the phase angles of the sinusoidal ac component, which is known from Fig. 1b and Fig. 3b,c. To improve the rapidity of protection, the sinusoidal ac component can be estimated in one period by the phase-domain trajectory of the short-circuit current.

As shown in Fig. 3b, the phase angles of point *c* and *d* approach the phase angle of point *h*. The average of the positions of point *c* (131.4°) and *d* (131°) is taken as the position of the trajectory circle corresponded to waveform of the sinusoidal ac component:

$${\theta }_{mk}=(131.4^\circ +131^\circ )/2=131.2^\circ$$

The initial phase angle of the sinusoidal ac component is calculated by Eq. (5), and it is:

$${\varphi }_{0k}=90^\circ -131.2^\circ =-41.2^\circ$$

The amplitude of the sinusoidal ac component is estimated by the average of the absolute values of the polar diameters of point *c* (7.36) and *d* (− 1.897), which is:

$${A}_{k}=(7.36+1.897)/2=4.6285$$

Then the sinusoidal ac component is estimated as:

22 $${i}_{p}{\prime}\left(t\right)=4.6285sin[100\pi \left(t-0.04\right)-41.2^\circ ], 0.04s\le t\le 0.06s$$

Obviously, the expression in Eq. (22) is more inaccurate than that in Eqs. (1) and (21).

### Errors in reformulated waveforms

From the relationship between Eqs. (1) and (2), when the phase-domain trajectory of a sampled waveform in fault recording is plotted, the polar diameter of each sample point is the instantaneous value of the waveform, and the polar angle of each sample point—its phase angle. In order to guarantee the correspondence between a waveform and its trajectory, it is inevitable to determine the 0-th sample point (SP) (the 0-th SP or SP0), where there is *t* = 0 and *θ* = 0°. This sample point is referred to as a reference one, and its phase angle is equal to the initial phase angle of the waveform.

The reference point can be any one of sample points, and it is usually the first one following a zero-crossing instant from negative to positive (or the reverse). Once the phase angle of the reference point, which is also SP0, is fixed, the phase angles of following SP1, SP2, … are all calculated from the initial phase angle (*φ*_0_) and the sample period (*T*_*s*_). Obviously, only one reference point is needed in a waveform, otherwise there appeared confusion during its processing, and correct results will not be obtained.

The phase angle corresponded to the time interval between any two sample points is defined as a phase window. For example, the phase angle corresponded to one power frequency period (360° or 2*π* radians) is described as a power frequency window, the phase angle corresponded to a half power frequency period (180° or *π* radians)—a half power frequency window, and the phase angle corresponded to one sample period in one power frequency period—a sample window.

Expressing the system frequency, sample frequency, and sample window as *f*_0_, *f*_*s*_, and Δ*φ*, the number of the sample points in a power frequency period is *f*_*s*_ / *f*_0_, and the value of Δ*φ* is calculated by the following equation:

23 $$\Delta \varphi =360^\circ /\left({f}_{s}/{f}_{0}\right)=360^\circ {f}_{0}/{f}_{s}$$

A power frequency period is fixed, which is 360° or 2*π* radians, however, the size of a sample window varies with the changes of the system frequency and the sample frequency (see Eq. (23)). When the sample frequency remains unchanged, the size of the sample window is needed to be adjusted with the change of the system frequency.

**Figure 8:**
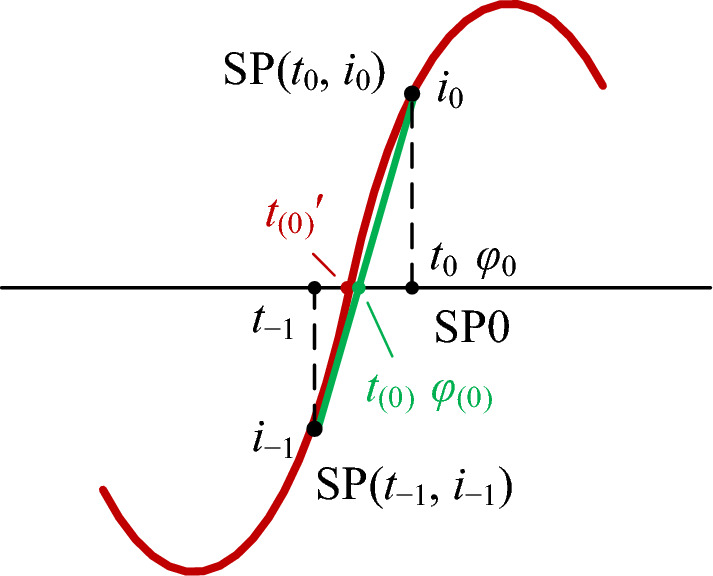
The figure demonstrates how to determine the phase angle of a reference point using the linear interpolation method, and how to estimate the calculated error in the phase angle of a sample point. The red curves are the sampled waveform of a current, and the green straight lines are formed by connecting the two sample points of the previous and subsequent the zero-crossing instant.

The zero-crossing instant from negative to positive of a current in Fig. 8 is *t*_(0)_ʹ. Current *i*_−1_ of the previous point (SP(*t*_−1_, *i*_−1_)) is negative, current *i*_0_ of subsequent point (SP(*t*_0_, *i*_0_)) is positive, and *t*_0_ − *t*_−1_ is the sample period (*T*_*s*_). SP(*t*_0_, *i*_0_) is the first positive sample point following the zero-crossing instant, and it is taken as a reference point (the 0-th SP or SP0).

Connecting SP(*t*_−1_, *i*_−1_) with SP(*t*_0_, *i*_0_) in Fig. 8. The figure demonstrates how to determine the phase angle of a reference point using the linear interpolation method, and to form a green straight line, its intersection (*t*_(0)_) with the time axis is approximated to the actual zero-crossing instant (*t*_(0)_′). When sample frequency *f*_*s*_ is much greater than system frequency *f*_0_, instant *t*_(0)_ is approximated to instant *t*_(0)_′. The value of *t*_(0)_ is calculated by a linear interpolation method, which is:

24 $${t}_{(0)}={t}_{-1}-{i}_{-1}\left({t}_{0}-{t}_{-1}\right)/\left({i}_{0}-{i}_{-1}\right)={t}_{-1}-{i}_{-1}{T}_{s}/\left({i}_{0}-{i}_{-1}\right)$$

The calculated value (*φ*_0*C*_) of the actual initial phase angle (*φ*_0_) is calculated by:

25 $${\varphi }_{0C}=\Delta \varphi \left({t}_{0}-{t}_{(0)}\right)/\left({t}_{0}-{t}_{-1}\right)=\Delta \varphi ({t}_{0}-{t}_{(0)}){f}_{s}$$

The calculated phase angle of the *n*-th sample point (SP(*t*_*n*_, *i*_*n*_)) *φ*_*nC*_ is calculated by the equation:

26 $${\varphi }_{nC}={\varphi }_{0C}+n\Delta \varphi$$

Since the calculated zero-crossing instant (*t*_(0)_) by Eq. (24) is not the actual one (*t*_(0)_ʹ), the error results in errors in *φ*_0*C*_ and *φ*_*nC*_ calculated by Eqs. (25) and (26). These errors are smaller when sample frequency *f*_*s*_ is much greater than system frequency *f*_0_.

As shown in Fig. 8, the error (*e*_*φ*_) in the calculated initial phase angle (*φ*_0*C*_) is:

$$e_{\varphi } = \varphi _{0} - \varphi _{{0C}} = \varphi _{{\left( 0 \right)}} = \Delta \varphi (t_{{(0)}} - t_{{(0)}}^{\prime } )f_{s}$$

Its percentage relative to the sample window is calculated by the following equation:

27 $${e}_{\varphi }\%={\varphi }_{(0)}/\Delta \varphi \times 100=({t}_{(0)}-t_{{(0)}}^{\prime }){f}_{s}\times 100$$

When sample frequency *f*_*s*_ = 20 000 Hz, actual zero-crossing instant *t*_(0)_ʹ = 0.004 674 559 726 073 8 s, and calculated zero-crossing instant *t*_(0)_ = 0.004 674 597 260 738 6 s, substituting them into Eq. (27), the error in the phase angles of the sample points is:

$${e}_{\varphi }\%=\left(0.004 674 597 260 738 6-0.004 674 559 726 073 8\right)\times 20 000\times 100=0.075 069 329 6$$

The larger the correlation coefficient between the reformulated waveform and its original one, the smaller the error in the reformulated waveform. The maximum correlation coefficient is 1, which means the original waveform can be completely restored from the reformulated one. However, due to the interference in the digitally sample waveforms in practice, the exact restoration of the original waveform is not contributed to the analysis of fault recording. The accuracy requirement is decided by the application scenarios of the results of fault recording analysis (“Supplementary information”).

## Conclusion

The research results show that the accuracy of reformulated fault recording is related to the features of the fault recording itself, the analysis purposes and the accuracy requirement. The algorithm studied in this article is not complex, where there are not time-consuming calculations, and the accuracy from the simulation data is relatively high. It is more reliable and understandable than other algorithms and easy to implement and spread in practice.

At present, the process analyzing and studying fault recording in a phase domain is still in an initial stage. In order to establish a theoretical basis, the simulated waveform in the article is used to achieve the algorithm. There is still a large gap between a simulated waveform and a practical one, and it is necessary to study them further in our subsequent researches. Let us make great efforts to work and look forward to it together.

### Supplementary Information

Supplementary Video 1.

Supplementary Information 1.

Supplementary Information 2.

## Data Availability

All data generated and analyzed during the current study are available from the corresponding author on reasonable request.
